# Combined Transcriptome and Proteome Analysis of Maize (*Zea mays* L.) Reveals A Complementary Profile in Response to Phosphate Deficiency

**DOI:** 10.3390/cimb43020081

**Published:** 2021-09-13

**Authors:** Zhi Nie, Bowen Luo, Xiao Zhang, Ling Wu, Dan Liu, Jialei Guo, Xuan He, Duojiang Gao, Shiqiang Gao, Shibin Gao

**Affiliations:** 1 Maize Research Institute, Sichuan Agricultural University, Chengdu 625014, China; maizemonom@126.com (Z.N.); bowen_maize@sicau.edu.cn (B.L.); hunterzap@163.com (X.Z.); wtl.570@163.com (L.W.); liu2883508@126.com (D.L.); wenpdsaas@126.com (J.G.); junssaas@126.com (X.H.); liyxsaas@126.com (D.G.); guirysaas@126.com (S.G.); 2 Institute of Biotechnology and Nuclear Technology, Sichuan Academy of Agricultural Sciences, Chengdu 610061, China

**Keywords:** maize, phosphate deficiency, transcriptome, proteome

## Abstract

A deficiency in the macronutrient phosphate (Pi) brings about various changes in plants at the morphological, physiological and molecular levels. However, the molecular mechanism for regulating Pi homeostasis in response to low-Pi remains poorly understood, particularly in maize (*Zea mays* L.), which is a staple crop and requires massive amounts of Pi. Therefore, in this study, we performed expression profiling of the shoots and roots of maize seedlings with Pi-tolerant genotype at both the transcriptomic and proteomic levels using RNA sequencing and isobaric tags for relative and absolute quantitation (iTRAQ). We identified 1944 differentially expressed transcripts and 340 differentially expressed proteins under low-Pi conditions. Most of the differentially expressed genes were clustered as regulators, such as transcription factors involved in the Pi signaling pathway at the transcript level. However, the more functional and metabolism-related genes showed expression changes at the protein level. Moreover, under low-Pi conditions, Pi transporters and phosphatases were specifically induced in the roots at both the transcript and protein levels, and increased amounts of mRNA and protein of two purple acid phosphatases (PAPs) and one UDP-sulfoquinovose synthase (SQD) were specifically detected in the roots. The new insights provided by this study will help to improve the P-utilization efficiency of maize.

## 1. Introduction

Phosphate (Pi) is a basic component of biomacromolecules, such as nucleic acids, proteins and phospholipids [1], is involved in cellular metabolism, signal transduction and photosynthesis [2], and plays a critical role in plant development, making it an essential macronutrient for plants. The high fixation rate of phosphorus (P) and slow diffusion of inorganic Pi in the soil mean that crops can use only 20–30% of the P fertilizer that is applied to arable soil [3], so continuous inputs of P fertilizer are necessary to sustain crop yields. However, sources of Pi rock are limited and will be used up in the coming century [4].

Plants have evolved complex regulatory signaling to cope with Pi limitation, an understanding of which will help us to address a Pi deficiency in agriculture. Recently, considerable progress has been made in discovering Pi homeostasis mechanisms in a range of species, including the enhanced efficiency of Pi acquisition and use [1]. Under Pi-deprived conditions, Pi acquisition is enhanced through the joint action of Pi transporters, the secretion of organic acids and phosphatases, and a change in root system architecture (RSA) [3]. Most high-affinity Pi transporter *PHT1* family members are induced in the root to promote Pi uptake in response to low-Pi [5,6], while the low-affinity Pi transporters in the *PHT2*, *PHT3*, *PHT4* and *PHT5* gene families are localized in organelles that are associated with Pi allocation within intracellular compartments [7,8,9,10]. Modification of the RSA is a critical trait in agriculture, with variation in the primary and lateral roots depending on the plant species [3]. In *Arabidopsis thaliana*, *PDR2* (encoding a P5 type ATPase) and *ALS3* (encoding an ABC transporter) cooperate with *LPR1/2* (encoding ferroxidases) to remodel the RSA under Pi deficiency [11,12]. During Pi homeostasis, a central regulator, *PHR1*, controls a large subset of Pi-responsive genes [13] and the PHR1–miR399–PHO2 signaling pathway plays a key role in the homeostasis process [14]. Furthermore, it has been shown that the SPX-domain proteins *SPX1*, *SPX2* and *SPX4* negatively regulate PHR to adapt to Pi starvation [15,16,17]. However, although much progress has been made in understanding Pi-responsive signaling in Arabidopsis and rice, we still have a poor understanding of this mechanism in maize (*Zea mays* L.), despite the economic importance of this crop.

High-throughput sequencing technology is an advanced method for the analysis of profile changes in transcripts and proteins due to its deep coverage, high resolution and dynamic landscapes [18]. However, although several studies have investigated the transcriptomes and proteomes of Arabidopsis [18] and soybean [19], few have considered maize. Therefore, in this study, we analyzed changes in the transcript and protein profiles of shoots and roots of the low-Pi-tolerant maize inbred line 178 under Pi deficiency using RNA sequencing (RNA-seq) and isobaric tags for relative and absolute quantitation (iTRAQ). This combined transcriptome and proteome analysis provides new information regarding the Pi-responsive mechanisms in the seedling stage of maize and expands our understanding of maize adaptation to Pi starvation.

## 2. Material and Methods

### 2.1. Plant Materials, Hydroponic Conditions and Measurement of Physiological Indexes

To identify the genes that are involved in the response to Pi starvation in maize, the Pi-efficient inbred line 178 was used as the research material in this study [20]. The maize seedlings were cultured using hydroponics following a previously described method [21] and were exposed to two Pi concentrations: control (CK, 1 mM KH_2_PO_4_) and low-Pi treatment (T, 1 μM KH_2_PO_4_).

Different tissues were harvested for physiological investigation. The shoots and roots were grown hydroponically and collected after 0 h, 24 h and 12 d to measure the biomass, S-P concentration and root to shoot ratio. The S-P concentration was measured by washing the fresh roots under running water for 20 min and then following a previously described method [22,23]. The root to shoot ratio was analyzed after drying the frozen shoots and roots (which had been frozen in liquid nitrogen) for 48 h by vacuum freeze drying (FreeZone^®^ 2.5 Liter Freeze Dry Systems, LABCONCO).

To investigate the root morphology, the roots were harvested at 12 d and the total length, total volume, average diameter and surface area were analyzed using the EPSON EXPRESSION 10000 XL root scanner and the WinRHIZO™ 2008a root analysis system.

### 2.2. RNA and Protein Extraction

Roots and shoots were harvested from plants in the T and CK groups at 12 d after treatment, with two independent biological replicates. The materials were homogenized in liquid nitrogen using a mortar and pestle and the total RNA was isolated using the TRIzol^®^Kit (Promega, Madison, WI, USA). RNase-free agarose gel electrophoresis was then performed using the Agilent 2100 Bioanalyzer (Agilent Technologies, Santa Clara, CA, USA) to verify the RNA quality (Figure S1). Protein extraction was performed using the TCA-acetone procedure [24] and the protein concentration was measured using the Bradford method.

### 2.3. RNA-seq Analysis

The total RNA was used for the RNA-seq analysis. First, a cDNA library was generated from the total RNA. Attached-oligo (dT) magnetic beads were then used to purify mRNA from the total RNA before fragmentation of the mRNA. ThecDNA library was performed on the Illumina sequencing platform (IlluminaHiSeq™ 2500) by Gene Denovo Co. (Guangzhou, China) with paired-end technology. A Perl program was written to select clean reads by removing low-quality sequences (>50% of bases had a quality of <20 in one sequence), reads with more than 5% N bases (bases unknown) and adaptor sequences were removed by a Perl program for screening clean reads. Sequencing reads in the FASTQ format were mapped to maize reference genome (B73 RefGen_V2) and splice junctions were identified using TopHat [25] with the following criteria: --GTF genome.g., tf, --no-coverage-search -r 80 --mate-std-dev 50. The Cufflinks [26] is a useful package for genome-guided transcript assembly and expression abundance estimation. For annotation analysis, National Center for Biotechnology Information non-redundant protein database (Nr) (http://www.ncbi.nlm.nih.gov/ (accessed on 12 October 2016)), KEGG (http://www.kegg.jp/ (accessed on 12 October 2016)) and GO (http://geneontology.org/ (accessed on 12 October 2016)) were used. Transcript expression was estimated as fragments per kilo base of transcript per million mapped reads (FPKM) with cuff quant and cuffnorm.

### 2.4. iTRAQ Analysis

For the iTRAQ analysis, 0.1 g of total protein from each sample was accurately prepared and then digested by incubating in a 20:1 solution of protein:trypsin for 4 h at 37 °C followed by a further 8 h in a fresh solution of protein:trypsin (20:1). After trypsin digestion, peptides were labeled with iTRAQ reagents as described previously [27], following which strong cation exchange (SCX) fractionation was performed. Finally, liquid chromatography-electrospray ionization-tandem mass spectrometry (LC-ESI-MS/MS) analysis was carried out using a Triple TOF^®^5600. The proteins were then simultaneously identified and quantified using Mascot software version 2.3.02 (Matrix Science), which only allows unique peptides to be chosen, providing more precise quantification. Searches were made against the maize database in Ensembl Plants (release 24) (39,469 sequences).

### 2.5. Enrichment Analysis

The web-based software agriGO (http://bioinfo.cau.edu.cn/agriGO/ (accessed on 12 October 2016)) was used to analyze GO enrichment [28]. The DETs and DEPs were mapped against the *Zea mays* L. AGPv3.30 reference genome using the Singular Enrichment Analysis tool with default settings. A *p*-value of ≤0.05 was used to identify significant GO terms.

### 2.6. Quantitative Real-Time PCR Validation

Quantitative real-time PCR was used to validate the deep sequencing results. Total RNA was extracted from the shoots and roots of inbred line 178 after 12 d of Pi treatment using the TRIzol Kit (Invitrogen, Waltham, MA, USA). Reverse transcription of mRNA was then performed using the PrimeScript™ RT Reagent Kit with gDNA Eraser (Perfect Real-Time) (TAKARA, Dalian, China) following the manufacturer’s instructions, with 1 μL of cDNA as the template in a 10-μL PCR reaction volume. Qua ntitative real-time PCR was performed with aCFX96™ Real-Time System (BIO-RAD) using RocheFastStartUniversalSYBRGreenMaster (Roche, Basel, Switzerland). Data were analyzed with the 2^−^^ΔΔCT^ relative quantification method [29] using the *myosin* as reference gene [30], and melt-curve analysis of target genes were showed in Figure S2. The primers for qRT-PCR were designed with Beacon Designer 7 software and showed in Table S1. 

### 2.7. Identification of Pi-Responsive TFs and Analysis of Protein–Protein Interactions

A list of TFs in maize was downloaded from three databases: PlnTFDB (http://plntfdb.bio.uni-potsdam.de/v3.0/ (accessed on 15 November 2017)) [31], GRASSIUS (http://www.grassius.org/ (accessed on 15 November 2017)) [32] and PlantTFDB (http://planttfdb.cbi.pku.edu.cn/ (accessed on 15 November 2017)) [33]. Only DEGs that could be found in all three databases at the same time were considered to be Pi-responsive TFs. Protein–protein analysis of Pi-responsive TFs was performed with the online website database STRING (http://string-db.org/newstring_cgi/show_input_page.pl?UserId=pZ3dhw_yaZK0&sessionId=1FcxNTFc8w6c (accessed on 1 March 2019)) [34] using a combined score of >700 as the criterion.

## 3. Results

### 3.1. Changes in Phenotypes and Soluble P Concentrations under Pi Starvation

**Figure 1:**
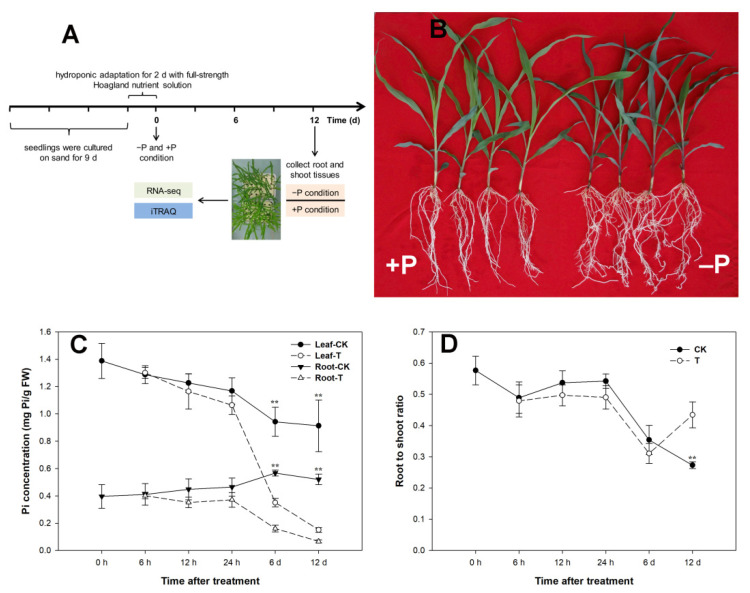
Phenotype analysis of 178 under Pi deficiency. (**A**) Schematic of the material preparation for transcriptomic and proteomic analysis. (**B**) Low Pi stimulated root growth and accumulated anthocyanin in shoots. (**C**) Pi concentration was measured in different tissues. (**D**) Root to shoot ratio analysis between control and low Pi treatment. Asterisks displays statistically significant analysis with Student’s *t*-test, ** represent *p* < 0.01.

To recognize the Pi-starvation-responsive genes in maize, seedlings of inbred line 178, which has a high tolerance for Pi deficiency, were exposed to two Pi concentrations: control (CK, 1 mmol/L KH_2_PO_4_) and low-Pi treatment (T, 1 μmol/L KH_2_PO_4_) (Figure 1A). After 12 days of growth, seedlings in the CK and T groups showed significantly different phenotypes. Under limited Pi conditions, seedlings exhibited a dwarf phenotype and were darker in color compared with the CK group (Figure 1B). In addition, Pi deprivation reduced the concentration of soluble P (S-P) (Figure 1C) and the biomass of the shoots and roots but increased the root to shoot ratio (Figure 1D), with these changes becoming particularly marked after 12 d. The total volume and surface area of the roots significantly increased after 6 days with the low-Pi treatment (Figure S3), increasing the ability for Pi absorption. These results confirm existing research data [35,36,37].

The concentration of S-P in the tissues indicates the growth status of a plant. We found that the concentration of S-P was much higher in the shoots than in the roots under both the CK and T conditions (Figure 1C). It is known that plants acquire Pi from the soil and the roots area unique organ for absorbing S-P from the soil, so the roots can be considered Pi sinks in plants, from which the S-P are transferred to every cell via the complex vascular tissue. In the seedling stage, more S-P is needed to meet the demand of the rapidly growing shoots, so the concentration of S-P is higher in the shoots than the roots (Figure 1C). However, as the seedling continues to grow, the concentration of S-P reaches a balance between the shoots and roots. The concentration of S-P exhibited little change following the short-term (0–24 h) treatment with low-Pi but rapidly decreased after 24 h to 6 d of treatment, particularly in the shoots (Figure 1C).

### 3.2. Differentially Expressed Transcripts under Pi Starvation

To identify Pi-responsive transcripts in maize, total RNA was extracted from the shoots and roots after 12-d growth under the CK and T conditions using two biological replicates. RNA was used in the transcriptome sequencing and eight RNA libraries were constructed: CS1 (CK, shoots, biological replicate 1), CS2 (CK, shoots, biological replicate 2), TS1 (T, shoots, biological replicate 1), TS2 (T, shoots, biological replicate 2), CR1 (CK, roots, biological replicate 1), CR2 (CK, roots, biological replicate 2), TR1 (T, roots, biological replicate 1) and TR2 (T, roots, biological replicate 2). The libraries were sequenced using Solexa high-throughput sequencing technology, giving a total of 38,708,766, 44,366,246, 38,377,062, 33,608,832, 51,365,972, 48,325,536, 44,288,312 and 39,318,954 raw reads, respectively (Table S2). The removal of useless tags from the raw data left approximately 42,214,602 clean read tags for each sample. Approximately 70.55–80.21% of the unique reads were perfectly mapped to the maize genome (B73 RefGen_V2) and 32 146 known genes were identified (Table S2).

**Figure 2:**
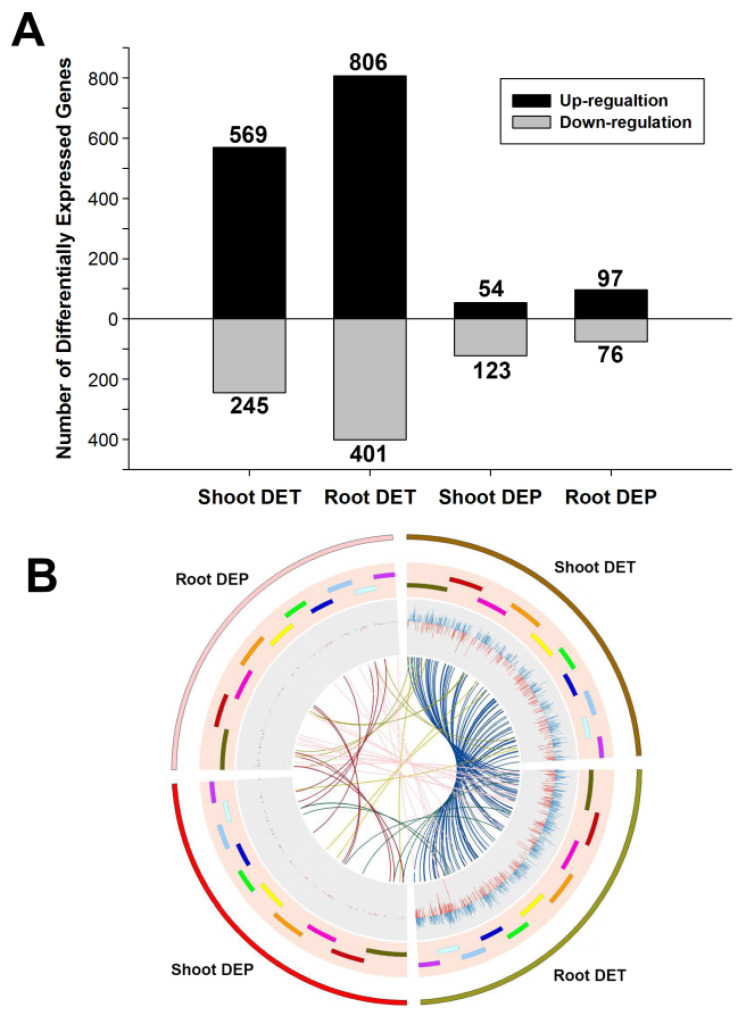
Identification of differentially expressed genes. (**A**) DETs and DEPs in roots and shoots. (**B**) Combination analysis of DET and DEP, from the outside to the inside, the outermost circle represents different differentially expressed genes. The penultimate circle represents the chromosome of maize. The third last round represents the fold change in gene expression. The lines in innermost represent genes showed differential expression at different tissues or different expression levels.

In this study, differentially expressed genes were identified using a threshold *p*-value of ≤0.05 and an absolute value of log_2_Ratio ≥ 1. A total of 1944 differentially expressed transcripts (DETs) were identified, of which 737 were uniquely expressed in the shoots, 1130 were uniquely expressed in the roots and 77 were expressed in both the shoots and roots under Pi starvation (Figure 2A, Table S3). There were more up-regulated DETs than down-regulated DETs (Figure 2B). To verify the differently expressed genes, the expression profiles of 17 DETs were analyzed using quantitative reverse transcription-polymerase chain reaction (qRT-PCR). This showed that there was an excellent relationship between the qRT-PCR and RNA-seq data (Figure S4), indicating that the RNA-seq data were reliable.

To gain an insight into changes in the functional categories under low-Pi conditions, the Gene Ontology (GO) categories of the 1944 DETs were analyzed using agriGO. In the shoots, the mitochondrion (34 genes) was the cellular component with the highest abundance, catalytic activity (237 genes) was the major term for molecular function and phosphate metabolic process (60 genes) and phosphorus metabolic process (60 genes) were the most significant terms in the biological process category (Table S4), indicating that a group of DETs was involved in the low-Pi-responsive mechanism. In the roots, different GO categories of DETs showed enrichment, with 76 DETs being enriched in the intrinsic to membrane GO term and 74 being enriched in the integral to membrane GO term (Table S4), revealing that a group of genes located in the membrane changed their expression profile to sustain the cells under Pi deficiency. Furthermore, catalytic activity (359 genes) was the most significant category for molecular function and genes belonging to the response to stimulus (118 genes), response to stress (83 genes) and oxidation reduction (80 genes) categories were the most redundant terms (Table S4).

### 3.3. Differentially Expressed Proteins under Pi Starvation

To identify the expression of proteins under Pi starvation in maize, a proteome analysis was performed on the same samples that were used for transcriptome analysis. iTRAQ was performed to identify the differentially expressed proteins (DEPs) in response to low-Pi. A total of 340 DEPs were recognized, among which 167 were uniquely expressed in the shoots, 163 were uniquely expressed in the roots and 10 showed differential expression in both the shoots and roots (Figure 2A). Interestingly, the proportion of proteins that were up- and down-regulated differed between the shoots and the roots, with more proteins being down-regulated than up-regulated in the shoots and the reverse pattern being observed in the roots (Figure 2A). This result is consistent with the dwarf phenotype of maize shoots that was observed under Pi starvation (Figure 1B), as protein synthesis decreased in the shoots to maintain the basic needs for plant growth and development, while the biomass of the roots increased to improve the ability to locate and acquire more Pi to meet the requirements of the plant.

The DEP profile was also very different between the shoots and the roots. In the shoots, the fold change in DEPs ranged from −1.66 to 1.99 (Table S3), with 123 proteins being down-regulated and 54 being up-regulated (Figure 2A). By contrast, in the roots, there was a wider variation in the change in abundance of DEPs, ranging from −2.6 to 3.5 (Table S3), and more proteins were up-regulated by low-Pi, with 97 proteins being induced and 76 decreasing (Figure 2A). In addition, most DEPs differed between the shoots and the roots under low-Pi, with only 10 proteins showing differential expression in both organs at the same time (Figure 2B). Annotation analysis of the DEPs in the roots and shoots showed that a subset of ribosomal proteins showed drastic changes in the shoots and it is noteworthy that all of these were down-regulated by Pi starvation (Table S3). It is supposed that the synthesis of proteins was dramatically reduced to meet the Pi requirements of the roots under low-Pi.

In contrast with the DETs, there were distinct differences in the enriched GO terms of the DEPs in the shoots and roots. In the shoots, the components of cell parts (149 genes) were overrepresented among the GO terms, and nucleotide binding (49 genes) and structural constituent of ribosome (47 genes) were enriched in the molecular function category (Table S4), with most DEPs that belonged to these terms being down-regulated under Pi deficiency (Table S3). This indicates that synthesis metabolism is suppressed in maize shoots under low-Pi conditions. Furthermore, 141 DEPs were significantly overrepresented in the metabolic process category (Table S4). By contrast, in the roots, catalytic activity was overrepresented among the GO terms (Table S4), which is consistent with the transcriptome data, and the response to stimulus and stress processes was also enriched (Table S4), which may indicate a response to Pi starvation at the post-transcriptional level.

### 3.4. Relationship between Differentially Expressed Transcripts and Proteins

Regulation at the transcriptional level will usually affect the final protein. However, the amount of protein can also be regulated at the post-transcriptional and translational levels–for example, miRNA may inhibit translation of the protein and the degradation of sub-cellular compartments [38]. Therefore, to identify the relationship between differentially expressed genes (DEGs) at the transcript and protein levels, we compared the DEGs that were identified in the transcriptome and proteome data.

In shoots of inbred line 178, four genes showed different expression profiles at both the transcript and protein levels under Pi deficiency and exhibited the same regulation trends (Table S3). Both the transcript and protein of trehalase (GRMZM2G162690) were up-regulated at the same time, and trehalase is thought to be involved in the defense mechanism in high plants, which would include a response to low-Pi since this is an important abiotic factor in the environment. Moreover, both the transcript and protein of ascorbate peroxidase (GRMZM2G054300) significantly increased under Pi deficiency.

By contrast, 23 genes were regulated simultaneously at the transcript and protein levels in the roots. Five genes exhibited reduced transcript and protein expression at the same time, all of which are involved in the carbon mechanism and oxidation reduction process. Meanwhile, 16 genes were up-regulated at the transcript and protein levels. Three isoforms of acid phosphatases (GRMZM2G315848, GRMZM2G093101 and GRMZM2G152447) sharply increased in abundance at both the transcript and protein levels and it is known that plants secrete acid phosphatase to acquire more soluble Pi under Pi deficiency. By contrast, the transcript and protein expression of aquaporin (GRMZM2G168439) decreased under Pi deficiency, which is consistent with the findings of many previous studies on plant nutrient deficiency [39]. In addition, another isoform of ascorbate peroxidase (GRMZM2G137839) was also found to be up-regulated by low-Pi at the transcript and protein levels, indicating that the oxidation process is very important for Pi homeostasis in the shoots and roots of maize. It is known that the expression level of SQD is specifically up-regulated by Pi starvation in plants [40,41,42], and here, SQD (GRMZM2G053322) not only increased at the transcript level but also at the protein level in the roots of inbred line 178, indicating that it is mainly regulated at the transcriptional level under low-Pi conditions in maize.

### 3.5. Low-Pi Induced Genes

Many studies have investigated the mechanism of Pi homeostasis in plants. However, most have focused on Arabidopsis as a target, with little being known about maize, despite this being a major crop globally. We found that a number of genes changed their expression at the transcript and protein levels under low-Pi conditions. Functional genes and regulators work together to contribute to plant growth and development, and low P stimulates groups of functional genes in plants. Under Pi deficiency, a set of functional genes that is associated with Pi acquisition and re-localization experience changes in their expression profiles, with Pi transporters being particularly greatly influenced. Five Pi transporters (PHTs) experienced increased expression at the transcript and protein levels (Table 1). At the transcript level, three PHTs sharply increased in the roots and one moderately increased in the shoots, while at the protein level, two PHTs experienced enhanced expression only in the roots. Notably, none of these PHTs exhibited increased expression at both the transcript and protein levels in the same tissue. These findings indicate the diverse regulation mechanism of PHT in response to Pi starvation. The organic form of total P ranges from 30% to 70% in arable soil [3], so the secretion of enzymes is important for releasing Pi from this store. In the present study, we identified seven acid phosphatases, two purple acid phosphatases and one nuclease (Table 1), the encoding genes for which all exhibited enhanced expression under low-Pi conditions. Furthermore, four of these showed increased expression at both the transcript and protein levels at the same time in the roots, indicating that these phosphatases play a critical role in the response of maize to Pi deprivation.

Regulation of the root architecture is a major strategy for responding to Pi starvation and auxins play a critical role in this process. In this case, 10 auxin-related genes were induced by low-Pi in our study (Table 1), with two *ZmPIN* genes being identified in the roots and one being identified in the shoots in response to Pi starvation. This indicates that the auxin signaling pathway is involved in the maize Pi-responsive induction pathway. Under Pi limitation, sulfolipids will replace phospholipids in the membrane to perform the same function and two predicted SQDs were greatly induced in maize (Table 1), among which the protein expression of GRMZM2G053322 was enhanced dramatically.

**Figure 3:**
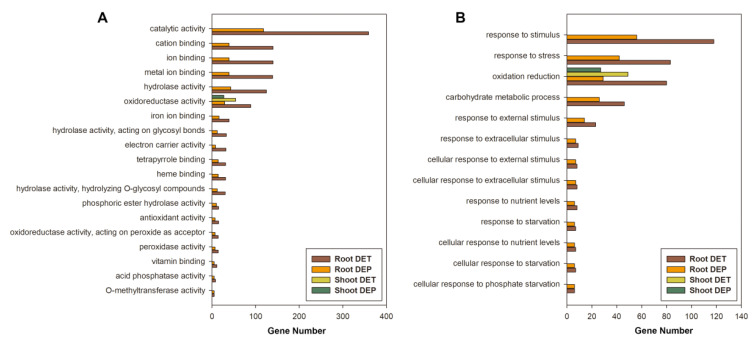
GO terms enrichment comparison between DETs and DEPs. GO enrichment analysis of DETs and DEPs and displayed on Molecular function (**A**) and biological process (**B**).

Comparison of the significantly enriched GO terms in the transcriptome and proteome data showed that genes that control biological processes associated with responses to deficiencies in Pi and other nutrients were overrepresented at the transcript level in maize shoots and roots (Figure 3B) and genes related to oxidation mechanisms were enriched in the shoots and roots at both the transcript and protein levels (Figure 3). Thus, it seems that the response to low-Pi focuses on transcript regulation.

### 3.6. Landscape of the Interaction between DEGs and Transcription Factors

**Figure 4:**
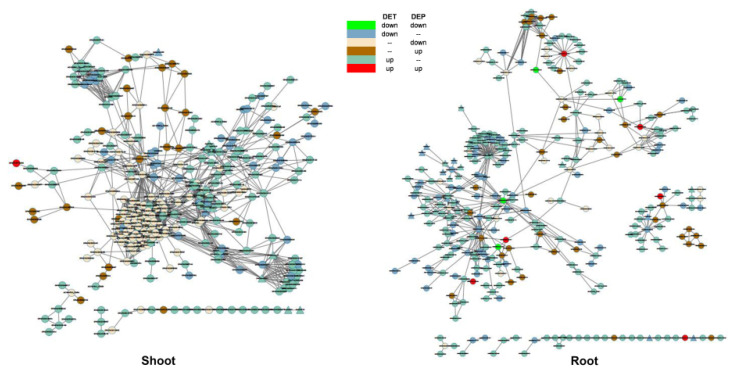
Protein interactions of differentially expressed genes in shoot and root.

It is known that many functional partnerships and interactions occur between proteins that are involved in central cellular processing [34]. The STRING database provides a useful opportunity for investigating the relationship between DEGs during the Pi homeostasis process in maize [34]. In the shoots, 987 DEGs were observed at the transcript and protein levels (Table S3), 299 of which were predicted to have interactive relationships (Figure 4). The core relationship among DEGs was in the interaction of ribosomal proteins (Figure 4), indicating that transcriptional and translational regulation occurs in maize shoots under low-Pi. In the roots, 309 of 1358 DEGs were found to be involved in this interactive relationship (Figure 4), with six DEGs showing up-regulation at both the transcript and protein levels in this network.

Among these DEGs, SQD (GRMZM2G053322) sharply increased. Under low-Pi, the translocation of sugar leads to changes in the root to shoot ratio and SQD plays an important role in this metabolic process. Moreover, it was found that this enzyme is located in the center of the protein–protein interaction of carbon homeostasis under Pi deficiency in maize (Figure 4). Five other genes also showed up-regulated expression at both the transcript and protein levels in this network (Table S3, Figure 4), among which the multifunctional protein gene GRMZM2G117357 can interact with 16 other DEGs (Figure 4), including a subset of genes that are involved in the oxidation process, indicating that oxidation is changed to adapt to low-Pi conditions.

Transcription factors (TFs) play a pivotal role in the regulatory mechanisms of plants and in the present study, 145 TFs showed differential expression at the transcript or protein level (Table S5). This included MYB, WRKY, ARF and bHLH, which particularly showed changes at the transcript level. These TFs may play a role in the response to Pi starvation, and transcriptional regulation may contribute to the main mechanism of Pi homeostasis in maize.

## 4. Discussion

To investigate the dynamic changes in proteins and transcripts under Pi deficiency in maize, it is necessary to consider the cooperation between multiple regulatory processes. Most previous omic studies have focused on individual changes, such as the transcriptome, miRNAs and proteome [18,43]. However, our combined transcriptome and proteome analysis showed that a wide range of transcripts and proteins changed significantly under Pi starvation in maize and allowed any differences to be compared.

Plants have developed elaborate mechanisms to cope with Pi starvation stress, including a range of morphological and biochemical changes [44]. Among these, the ability to change the root morphology is a key component of the Pi starvation response strategy. Under low-Pi conditions, plants modify their root structure by altering cell growth to increase the absorption ability of the roots. Previous studies on maize have shown that shallower root systems that have more lateral roots are better adapted to Pi deficiency [45]. Similarly, our results showed that inbred line 178 of maize, which is Pi-tolerant, developed more lateral roots and a higher root to shoot ratio under low-Pi conditions (Figure 1), which is consistent with the findings of previous studies [46]. In Arabidopsis, Pi limitation stimulates an increase in Pi transporters and phosphatases for the formation of lateral roots and root hairs [3]. However, maize appears to have a more complex mechanism.

The root development mechanism is very complex in plants and the regulation of root growth under Pi deficiency is mediated by many factors, such as auxin signaling and expansion [47]. It is known that the RSA is regulated by the auxin gradient [48,49] and several regulators and auxin transporters were found to be involved in the Pi homeostasis pathway in maize. As shown in Table 1, two putative *ZmPINs* exhibited increased transcript expression in the roots under Pi starvation, whereas these could hardly be detected under normal Pi conditions (Table S3), as found for Arabidopsis [50]. However, while *AtPINs* showed minor changes during the early stages of Pi starvation in Arabidopsis, we found that *ZmPINs* dramatically increased in the roots of maize, possibly due to differences in the responses of the seedlings between the early and later periods of exposure to Pi deficiency.

In shoots, Pi deficiency leads to a decrease in photosynthesis. The eukaryotic ribosome is a complex structure that is composed of four ribosomal RNAs (rRNAs) and approximately 80 ribosomal proteins and represents an essential piece of the cell machinery, being responsible for protein synthesis and consequently playing a major role in controlling cell growth, division and development [51]. In our study, a number of ribosomes experienced reduced expression at the protein level but not the transcript level (Table S3). By contrast, while previous studies have also shown that ribosomal proteins decrease under Pi deficiency, this was at the transcript level [52].

The alternation of P recycling is another strategy that plants can use to respond to Pi deficiency. One of the largest pools of P in plants is found in the phospholipids, which are key components of cell membranes. Consequently, increasing the mobilization and recycling of Pi, such as through the hydrolysis of phospholipids, are important strategies for providing enough Pi to meet the growth requirements of plants [2]. Here, we found that SQD (GRMZM2G053322) increased at both the transcript and protein levels in the roots to improve the Pi content and thus maintain plant growth. Furthermore, some Pi transporters and phosphatases were also induced in the roots and shoots of inbred line 178 at both the transcript and protein levels.

A comparison of the transcriptomes and proteomes of the shoots and roots of maize indicated that there were marked differences between these, allowing us to explore the potential mechanisms of Pi homeostasis in this important crop. At the transcript level, most of the DETs were clustered as regulators, such as TFs, and DETs that are associated with the Pi signaling pathway were significantly enriched in both the shoots and roots. However, at the protein level, expression changes were more often observed in functional genes and metabolism-related genes. This weak correlation between the transcript abundance and protein expression profile indicates that post-transcriptional regulation is important for Pi homeostasis in maize. Our results highlight the changes in transcriptome and proteome regulation signaling that occur in response to Pi starvation and provide new insight into the transcriptional and post-transcriptional regulation mechanisms in maize.

## 5. Conclusions

In this study, the key goal was to identify the candidate genes involved in the Pi deficiency responding mechanism in maize. A multiple-level analysis in transcript and protein expression changes with RNA-seq and iTRAQ was performed, and Low-Pi induced 1944 DETs and 340 DEPs in the seedling of maize. In addition, most of regulators changed expression in transcript level, whereas functional genes converted protein expression level. The integrated analysis of DET and DEP revealed that only a few genes change expression at both the transcript and protein levels, such as Pi transporters and phosphatases. Moreover, two PAPs and one SQD were specifically induced under Pi deficiency in roots of seedling of maize. These findings have disclosed a complimentary profile in response to Pi deficiency in maize, providing new clues to explore the molecular mechanism in response of maize to Pi deficiency.

## Figures and Tables

**Table 1:** Low-Pi induced genes.

Gene_ID	RNA-seq		iTRAQ		
	log2_FC (TL/CL)	log2_FC (TR/CR)	log2_FC (TL/CL)	log2_FC (TR/CR)	Description
GRMZM2G009800	--	11.80	--	--	Inorganic phosphate transporter
GRMZM2G154090	3.62	--	--	1.88	Pht1;1
GRMZM2G326707	--	--	--	0.93	Pht1;2
GRMZM2G041595	--	12.38	--	--	Pht1;5
GRMZM5G881088	--	9.79	--	--	Pht1;6
AC202435.3_FG003	--	7.38	--	2.41	acid phosphatase
GRMZM2G014193	--	2.94	--	--	Chloroplast purple acid phosphatase
GRMZM2G093101	5.25	5.20	--	1.56	acid phosphatase
GRMZM2G109071	5.97	--	--	--	acid phosphatase
GRMZM2G134054	--	5.54	--	--	acid phosphatase
GRMZM2G141584	--	4.79	--	--	acid phosphatase
GRMZM2G152447	--	8.50	--	3.49	acid phosphatase
GRMZM2G152477	3.10	--	--	1.13	Purple acid phosphatase 1
GRMZM2G315848	--	4.67	0.68	1.46	Nucleotide pyrophosphatase/phosphodiesterase
GRMZM2G015908	--	8.80	--	--	acid phosphatase
GRMZM2G100652	1.82	1.80	--	--	UDP-sulfoquinovose synthase (SQD2)
GRMZM2G053322	--	3.54	1.05	1.38	UDP-sulfoquinovose synthase
GRMZM2G175995	--	2.93	--	--	Auxin regulated gene involved in organ size 5
GRMZM2G354338	--	6.08	--	--	Auxin regulated gene involved in organ size 8
GRMZM2G082943	--	3.52	--	--	Auxin regulated gene involved in organ size 9
GRMZM2G429940	2.28	--	--	--	CBL-interacting serine/threonine-protein kinase 15
GRMZM2G059544	8.87	--	--	--	IAA25-auxin-responsive Aux/IAA family member
GRMZM2G126260	--	1.23	--	--	Putative auxin efflux carrier PIN10a
GRMZM2G025742	--	7.83	--	--	Putative auxin efflux carrier PIN5a
GRMZM2G040911	3.90	--	--	--	Putative auxin efflux carrier PIN5c
GRMZM2G074427	1.86	--	--	--	ZmIAA23
GRMZM2G045057	6.75	--	--	--	ZmLAX4

## Data Availability

The data presented in this study are available in this work.

## Supplementary Materials

The following are available online at https://www.mdpi.com/article/10.3390/cimb43020081/s1, Figure S1: RNA quality of samples for RNA-seq, Figure S2: Melt-curve analysis of target genes, Figure S3: Root phenotype analysis of 178 under Pi starvation, Figure S4: Validation of transcriptome data by qRT-PCR, Table S1: Gene list and primers for qRT-PCR, Table S2: Overview of RNA-seq sequencing data, Table S3: Identification of differentially expressed genes at the transcript and protein level, Table S4: Gene ontology (GO) enrichment analysis of DET and DEP, Table S5: Identificaito of differentially expressed transcription factors.
