# Supplementary figures and images for: Combined Transcriptome and Proteome Analysis of Maize (Zea mays L.) Reveals A Complementary Profile in Response to Phosphate Deficiency
Source: Curr Issues Mol Biol. 2021 Sep 13;43(2):1142–55. doi: 10.3390/cimb43020081 (PMC8929058; doi:10.3390/cimb43020081)

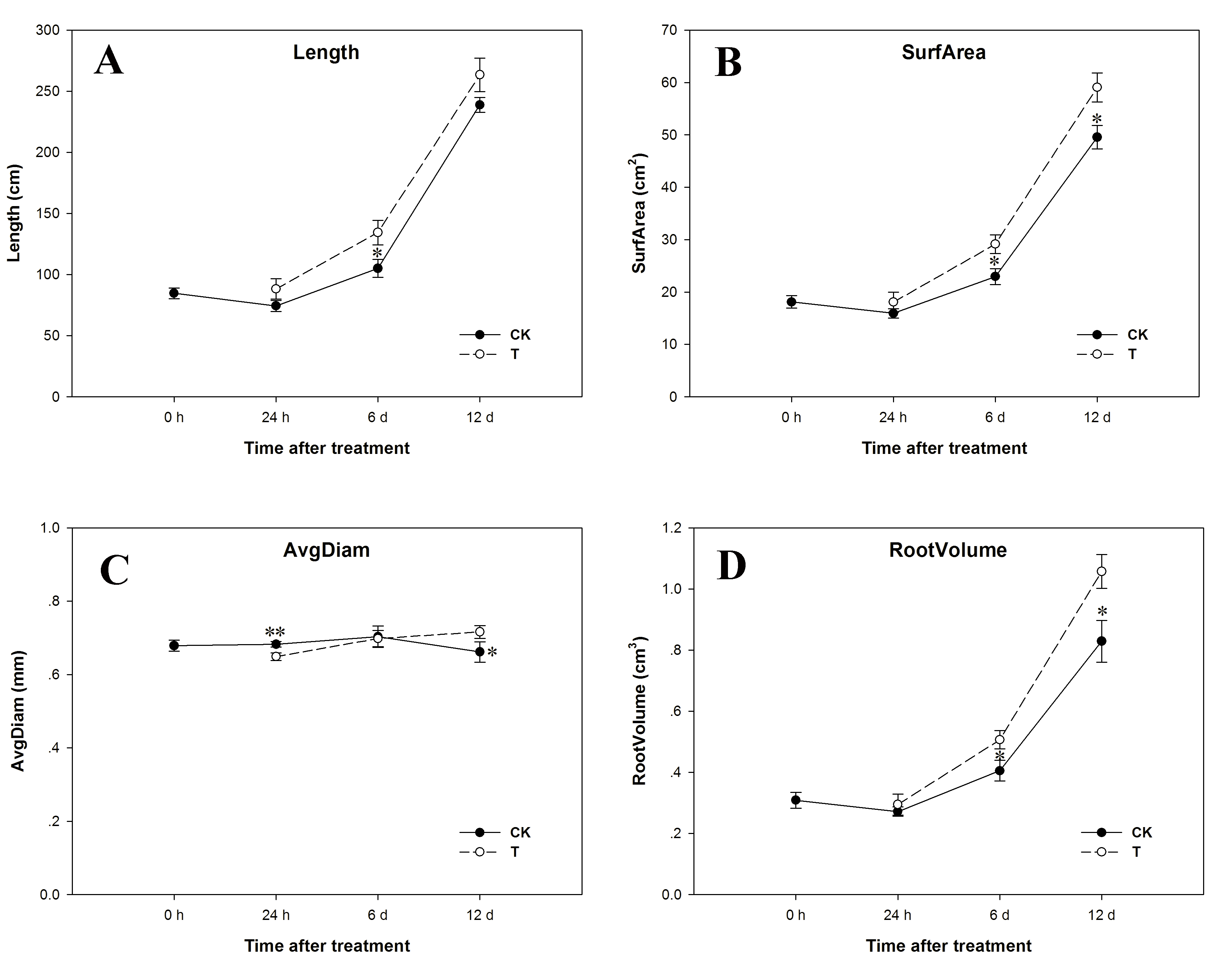

Supplement: Supplementary file 1 [file cimb-43-00081-s001.zip › Figure_S1.tif]

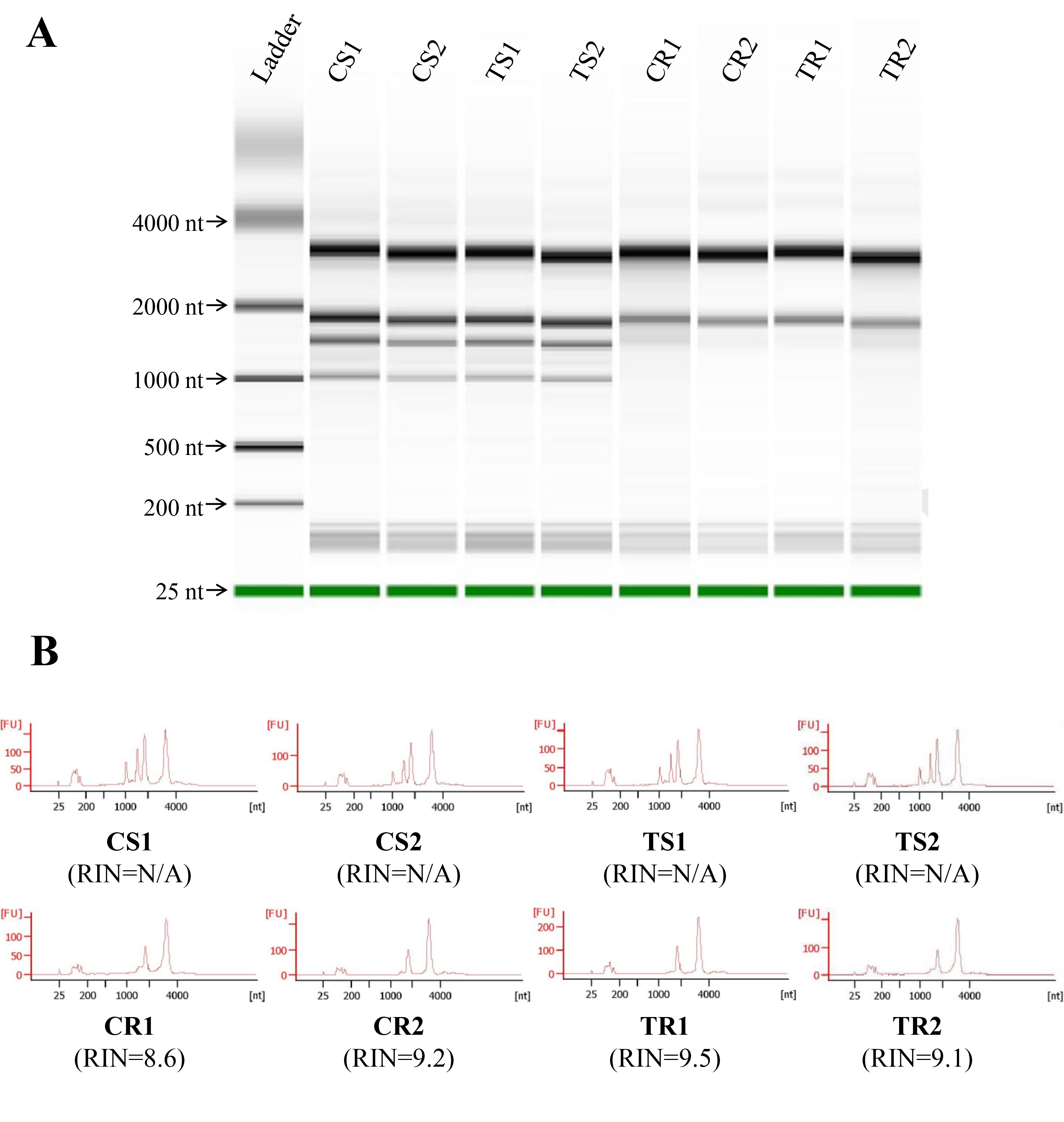

Supplement: Supplementary file 1 [file cimb-43-00081-s001.zip › Figure_S3.tif]

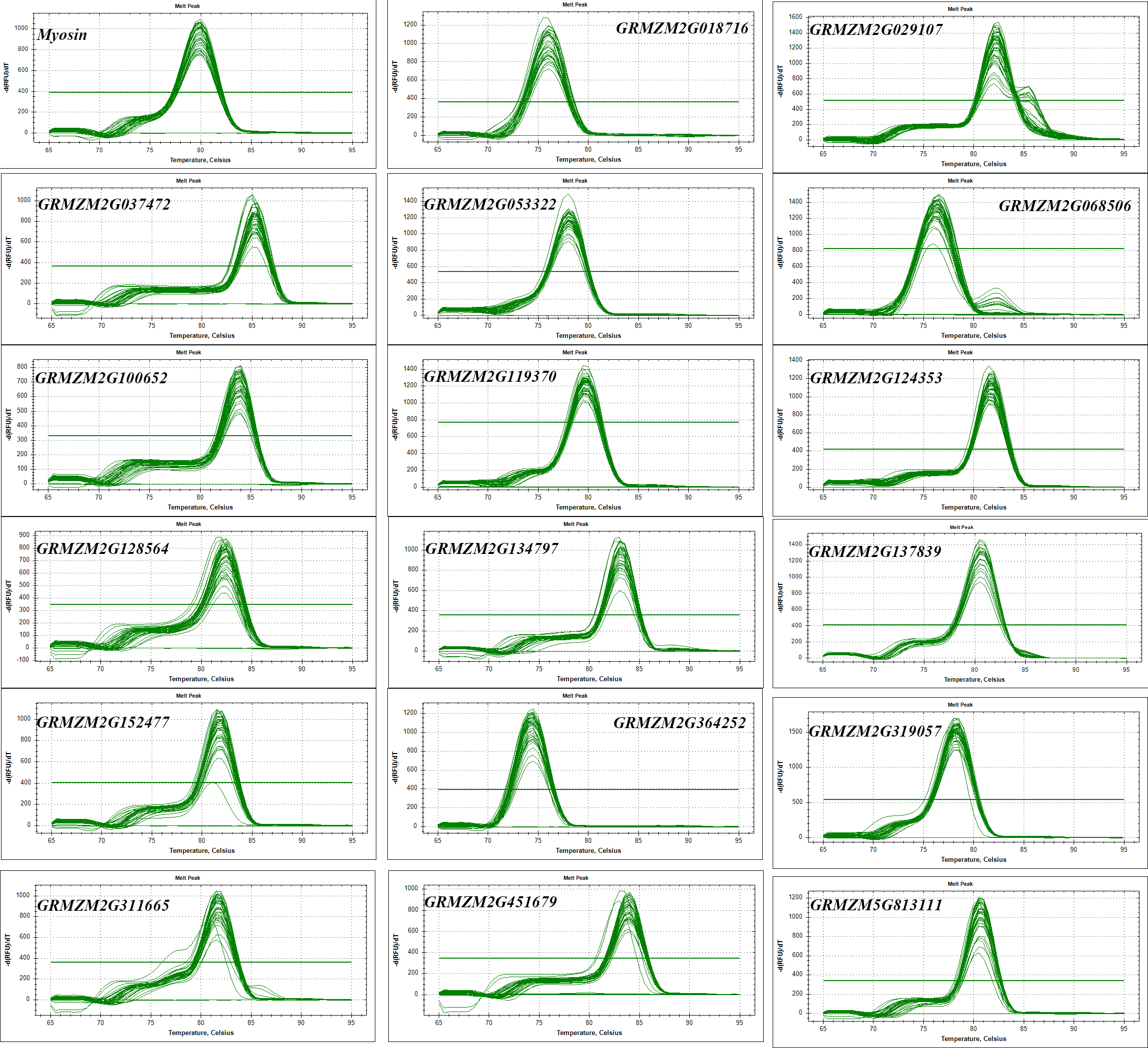

Supplement: Supplementary file 1 [file cimb-43-00081-s001.zip › Figure_S4.tif]
